# Novel Multifunctional Cannabidiol-Based Analogues with In Silico, In Vitro, and In Vivo Anti-SARS-CoV-2 Effect

**DOI:** 10.3390/ph18101565

**Published:** 2025-10-16

**Authors:** Graziella dos Reis Rosa Franco, Vanessa Silva Gontijo, Flávia Pereira Dias Viegas, Matheus de Freitas Silva, Cindy Juliet Cristancho Ortiz, Caio Miranda Damásio, Isabella Marie Fernandes Silva, Thâmara Gaspar Campos, Erik Vinicius de Sousa Reis, Felipe Alves Clarindo, Thaís de Fátima Silva Moraes, Matheus Müller Pereira da Silva, Patrícia Ribeiro de Carvalho França, Isabella Alvim Guedes, Laurent Emmanuel Dardenne, Jordana Grazziela Alves Coelho dos Reis, Patrícia Dias Fernandes, Claudio Viegas

**Affiliations:** 1 Laboratório de Pesquisa em Química Medicinal (PeQuiM), Instituto de Química, Universidade Federal de Alfenas, Alfenas 37130-001, Minas Gerais, Brasil; grazireisfranco@yahoo.com.br (G.d.R.R.F.); vanessagontijo@yahoo.com.br (V.S.G.); fpdviegas@gmail.com (F.P.D.V.); defreitassilva.matheus@gmail.com (M.d.F.S.); cindy.cristancho@gmail.com (C.J.C.O.); caiomdamasio@gmail.com (C.M.D.); isabela_mfsm@yahoo.com.br (I.M.F.S.); thgc2017@gmail.com (T.G.C.); 2 Laboratório de Virologia Básica e Aplicada, Instituto de Ciências Biológicas, Departamento de Microbiologia, Universidade Federal de Minas Gerais, Belo Horizonte 31270-901, Minas Gerais, Brasil; reis.erik@gmail.com (E.V.d.S.R.); fac3626@gmail.com (F.A.C.); thais.moraes00@hotmail.com (T.d.F.S.M.); reisjordana@gmail.com (J.G.A.C.d.R.); 3 Laboratório Nacional de Computação Científica (LNCC), Petrópolis 25651-075, Rio de Janeiro, Brasil; matheusp@posgrad.lncc.br (M.M.P.d.S.); isabella@posgrad.lncc.br (I.A.G.); dardenne@lncc.br (L.E.D.); 4 Laboratório de Farmacologia da Dor e da Inflamação, Instituto de Ciências Biomédicas, Universidade Federal do Rio de Janeiro, Rio de Janeiro 21941-590, Brasil; patriciaribeiro.ufrj@yahoo.com.br

**Keywords:** SARS-CoV-2, COVID-19, antiviral effect, ACE2 inhibitors, multifunctional cannabidiol-based analogues

## Abstract

**Background/Objectives**: COVID-19 was responsible for millions of deaths worldwide. This study aimed to identify substances with in vitro and in vivo effects against the SARS-CoV-2 virus. **Methods**: Compounds PQM-243 and PQM-249, two terpene-*N*-acyl-aryl-hydrazone analogues, were evaluated in vitro against SARS-CoV-2 to a antiviral activity and inhibitory effect against angiotensin converting enzyme 2 (ACE2). A possible inhibitory effect affecting the interaction between the receptor-binding domain (RBD) protein and/or ACE2 was evaluated using LUMMIT kit. A SARS-CoV-2-induced pulmonary pneumonia model was developed to evaluate the effects of the compounds after 3 days of treatment. **Results**: Compounds PQM-243 and PQM-249 exhibited IC_50_ values of 0.0648 ± 0.041 µM and 0.2860 ± 0.057 µM against SARS-CoV-2 with a selective index of >1543.21 and 349.65, respectively, and IC_50_ values of 12.1 nM and 13.3 nM, respectively, against ACE2. All concentrations used significantly reduced interactions between ACE2 and RBD. Computational studies suggest that these new compounds are potent direct anti-SARS-CoV-2 agents, capable of reducing both virus viability and its invasive ability in the host cells by reducing the interaction between RBD and ACE2. It was also demonstrated that even when administered by the oral route, both compounds reduced SARS-CoV-2-induced lung inflammation. Our data suggests that both compounds can act as potent direct anti-SARS-CoV-2 agents, reducing both viral viability and host cell entry. In addition, they exhibited a significant multi-target-directed pharmacological profile, also reducing SARS-CoV-2-induced lung inflammation when administered orally. **Conclusions**: Overall, these findings support further investigation of PQM-243 and PQM-249 as promising antiviral and anti-inflammatory multi-target prototypes for the development of innovative drug candidates targeting SARS-CoV-2 and other virus-related respiratory diseases.

## 1. Introduction

The COVID-19 pandemic, initiated in Wuhan (China) in December 2019, rapidly spread worldwide, causing millions of infections and deaths regardless of ethnicity, age, or socioeconomic status. By April 2020, over 1.2 million cases had been confirmed, and to date, the WHO reports more than 776 million cases and 7 million deaths, with the USA and Brazil among the most affected countries [1,2]. SARS-CoV-2 likely originated from animal coronaviruses that acquired the ability to infect humans. Genomic sequencing revealed a positive single-stranded RNA virus that uses its spike (S) protein to bind the ACE2 receptor, with cell entry facilitated by TMPRSS2 [3,4,5,6,7]. Once inside, the virus synthesizes polyproteins via RNA-dependent RNA polymerase, enabling replication and release of viral particles. Currently, the WHO recognizes eight circulating variants [8,9]. Understanding the viral life cycle has led to the identification of therapeutic targets, including non-structural proteins (PLpro, 3CLpro/Mpro, helicase, RdRp) and cellular entry mechanisms (ACE2, TMPRSS2, RPS3). The urgent search for treatments triggered large-scale drug repurposing efforts, with several antivirals (remdesivir, favipiravir, lopinavir–ritonavir, etc.) and natural products evaluated as entry inhibitors or immunomodulators [9,10,11,12,13,14,15]. Among these, remdesivir showed the best clinical outcomes, leading to FDA approval in 2021 [16,17,18,19,20,21,22,23,24,25,26]. The S protein remains the main therapeutic target, with approaches including neutralizing antibodies and the use of RBD-ACE2 blockers, fusion inhibitors, and protease inhibitors. Viral binding to ACE2 also disrupts the renin–angiotensin system (RAS), aggravating pneumonia, which highlights ACE2 and AT1R as therapeutic targets [27,28,29]. Some studies suggest ACE2 modulation could attenuate lung injury, as observed in animal models of acute lung injury (ALI) induced by LPS [30,31,32,33,34,35,36]. Severe COVID-19 is characterized by a hyperinflammatory state or “cytokine storm,” with IL-6 as a key mediator [37]. Anti-inflammatory agents such as IL-6 inhibitors (tocilizumab) and corticosteroids (dexamethasone) have shown benefits in reducing mortality in severe cases [21,27,29,38,39,40,41,42,43]. Thus, therapies combining antiviral activity and immunomodulation are crucial to control disease progression and prevent fatal complications.

**Scheme 1:**
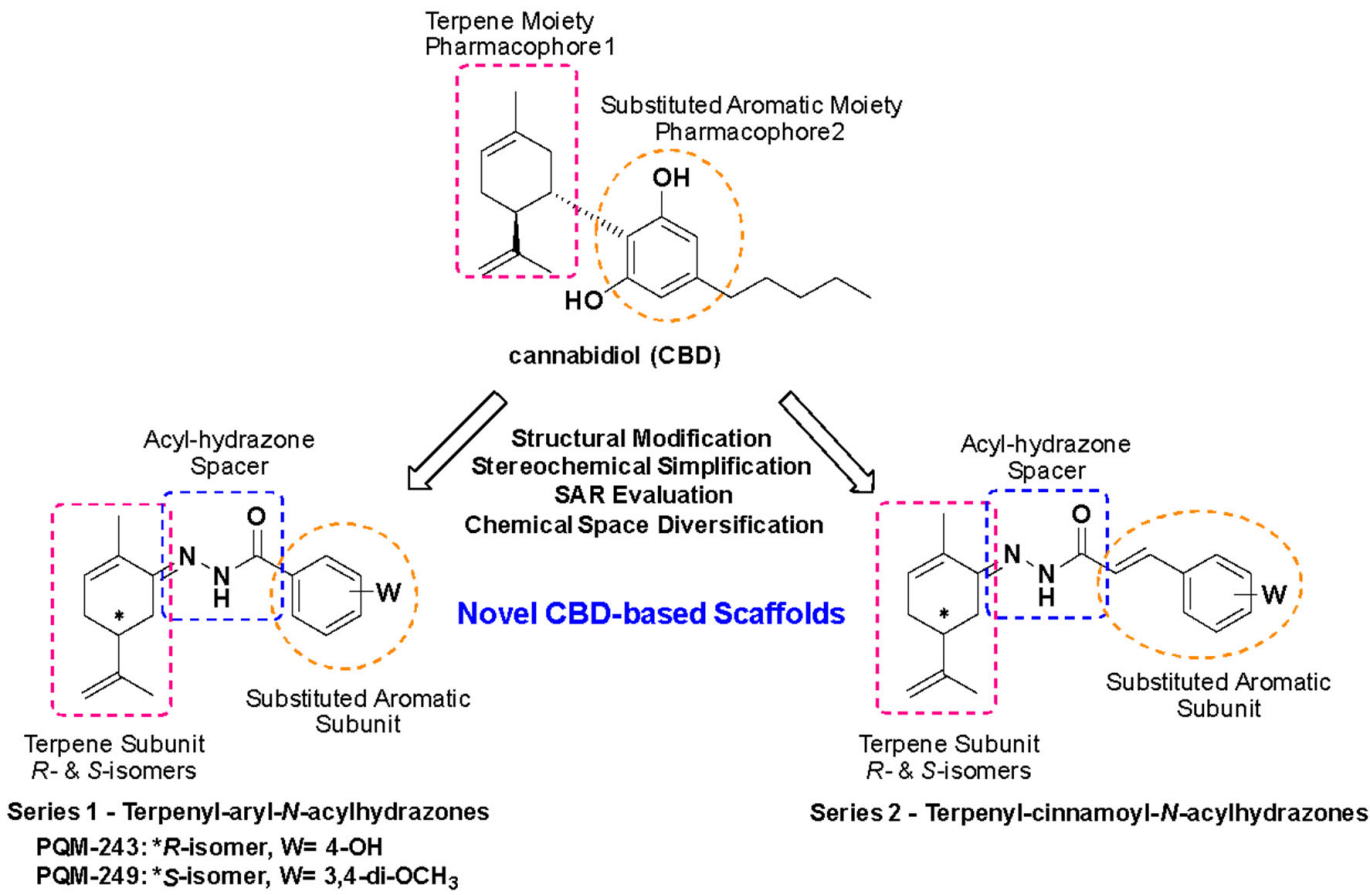
Rational design of two series of CBD-based N-acyl-hydrazones (series 1 and 2) and chemical structures of PQM-243 and PQM-249 with potential antiviral and anti-inflammatory effects against COVID-19.

This study aimed to investigate a chemical library of approximately 100 novel bioactive molecules, from different chemical classes, available at our Chemical Library at the Laboratory of Research in Medicinal Chemistry (PeQuiM) of the Federal University of Alfenas, Brazil. The primary goal was to identify substances capable of representing innovation in the treatment of pathophysiological manifestations associated with inflammatory processes in the respiratory system, with additional antiviral activity. The rationale behind selecting these substances was based on our recent results in different studies focused on several classes of new compounds with in vitro and in vivo anti-inflammatory, neuroprotective, antiparasitic, and antimicrobial effects [44,45,46,47,48,49]. Particularly, the study of two series of cannabidiol (CBD)-based terpene-*N*-acyl-aryl- (Series 1) and terpene-*N*-acyl-cinnamoyl (Series 2) hydrazones (Scheme 1), led to the identification of some compounds with intracellular antioxidant and anti-inflammatory effects, inhibiting the release of cytokines such as IL-1β and TNF-α. Primarily, these series were rationally designed as new CBD-based analogues, with a simplified structural pattern by abolishing one of the stereogenic centers in the terpene moiety of CBD, exploring a more diverse chemical space by introducing an *N*-acyl-hydrazone subunit as a linker between the substituted-aromatic and the terpene fragments, and analyzing the structure–activity contribution of distinct substituents on the aromatic ring. Moreover, the changes proposed in the structural architecture of the new CBD-based analogues could be important to address future modifications and the design of new CBD-based chemical entities, exploring diverse disposition of the terpene and aromatic fragments, as well as the position of linkage between these two structural subunits and the auxophoric contribution of different spacer fragments.

Among 28 of these CBD-based analogues, PQM-243 and PQM-249 exhibited the most promising in vitro anti-replicative effects against SARS-CoV-2 in preliminary screening assays and were selected for further investigation. Additionally, recent studies reported that CBD exhibited blocking effects on the replication of SARS-CoV-2 after viral entry, inhibiting viral gene expression and reversing many of its effects on host gene transcription. It was also evidenced that CBD inhibits SARS-CoV-2 replication, in part by up-regulating the host IRE1α ribonuclease endoplasmic reticulum (ER) stress response and interferon signaling pathways [50,51].

## 2. Results

### 2.1. Antiviral Activity

**Figure 1:**
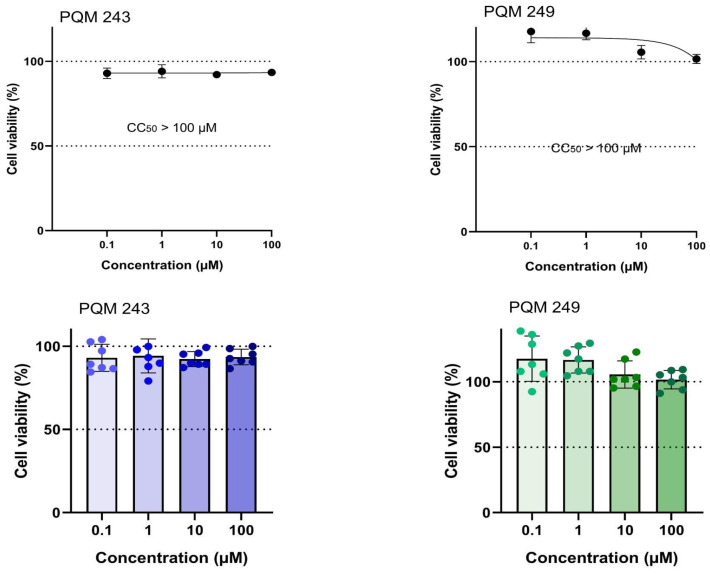
PQM-243 and PQM-249 did not show cytotoxicity against Vero Cells CCL-81. Four concentrations (0.1 to 100 μM) of PQM-243 and PQM-249 were incubated with the cells for 72 h, then alamarBlue™ reagent was added, and the results were measured by optical density using a spectrophotometer at 577 nm. Data are presented as mean ± standard error of the mean (SEM).Each column color represents one different concentration used.

The evaluation of the cytotoxicity of compounds PQM-243 and PQM-249 demonstrated that cell viability was not reduced from 85% for PQM-243 and was close to the 100% for PQM-249, even at the highest concentration of 100 μM and after 72 h of exposure to the cells (Figure 1). DMSO was used as a vehicle control at a concentration of 0.5% (*v*/*v*), which also did not interfere with cell viability. These results provided relevant data on the bioactivity of both compounds, particularly regarding the determination of the non-cytotoxic concentration, which was essential for establishing the therapeutic dose to be investigated.

**Figure 2:**
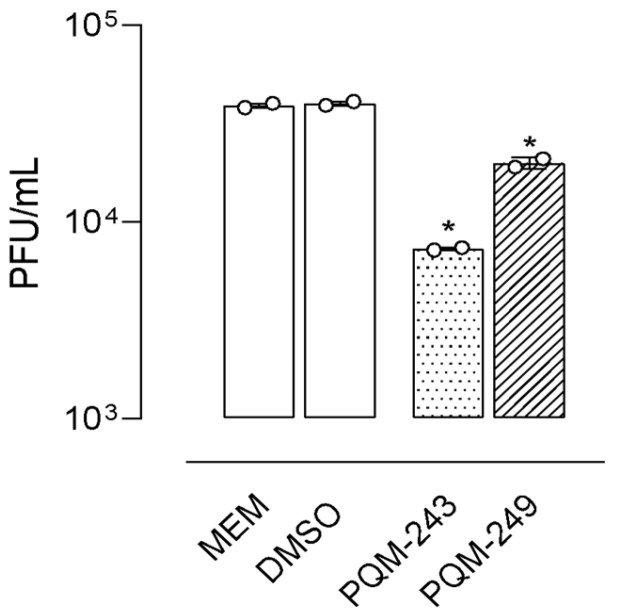
PQM-243 and -249 exhibited virucidal effects. The graphs display titration results after SARS-CoV-2 particles were incubated at 37 °C with PQM-243 and -249 (at 100 μM) for 1 h. Data are shown as mean ± standard error of the mean (SEM), * *p* < 0.05 indicates a statistical difference when compared to the viral control by ANOVA. MEM = minimal essential medium (viral control); PFU = plaque-forming units.

To evaluate their antiviral activity, compounds PQM-243 and PQM-249 were first tested against SARS-CoV-2 to determine whether they could reduce the viral load when in direct contact with the virus alone, prior to cell exposure. To this end, the virus and test compounds were incubated together for 1 h at 37 °C, after which the viral titer was determined. The viral titer graphs demonstrated that both compounds exhibited a significant virucidal effect. Comparative analysis between the cells infected with the untreated virus (MEM, cell control) and those infected with the virus pre-treated with PQM-243 and PQM-249 confirmed their ability to reduce virus infectivity, indicating a direct effect of the tested compounds on the viral particle. Notably, PQM-243 resulted in a 6-fold reduction in viral load, which was twice the effect observed for PQM-249, which exhibited a 3-fold reduction (Figure 2).

**Figure 3:**
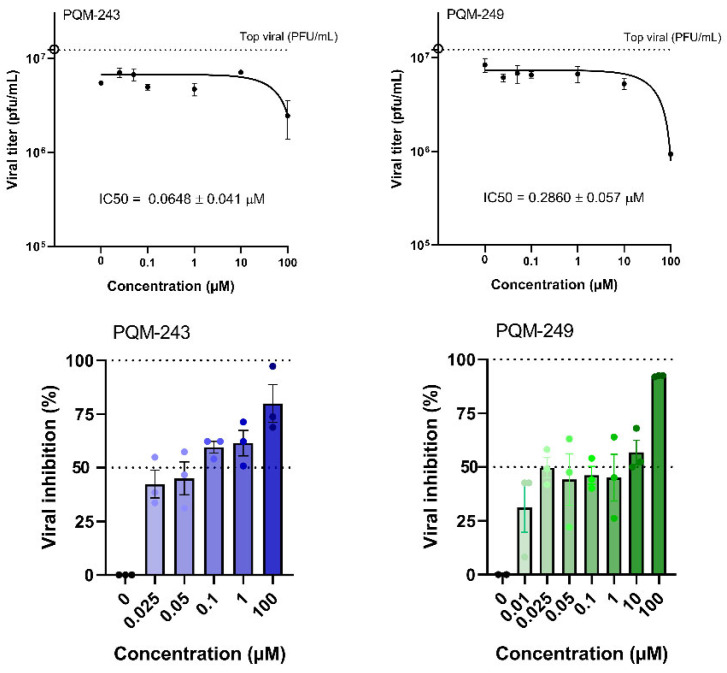
Dose–response curve and viral inhibition percentage graph of PQM-243 and PQM-249. Vero cells CCL-81 were infected with SARS-CoV-2. The median and interquartile range of the number of infective SARS-CoV-2 particles were assessed by the viral plaque assay. Trendlines obtained by logistic regression with the fourth logistic parameter are shown in the graphs. IC_50_ = half-maximal inhibitory concentration. Top viral = indicates the highest value of viral control titer (point with 0 µM of compound), Hollow circle in y axis = average of the viral control titer, used for IC_50_ calculation, PFU = plaque-forming units.

Next, the ability of PQM-243 and PQM-249 to reduce SARS-CoV-2 in infected cells after treatment was investigated. A cell monolayer was infected with SARS-CoV-2 (MOI 0.01), and after 1 h of adsorption at 37 °C, the cells were treated with each compound at different concentrations (0.1 to 100 µM) for 48 h. The viral load in the supernatant was then determined. The results confirmed the inhibition of viral infection, as a reduction in SARS-CoV-2 viral titer was observed in infected cells treated with both compounds (Figure 3). At concentrations of up to 1 μM, both compounds showed a significant reduction in viral titer. However, neither exhibited a dose-dependent response, as a similar reduction was observed at all tested concentrations.

Based on the values of relative inhibitory potency, the selectivity index (SI) was determined for each compound, an essential parameter for assessing the antiviral potential of a substance, while ensuring minimal cytotoxicity to host cells. Using CC50 and IC_50_ data, the SI values of PQM-243 and PQM-249 against SARS-CoV-2 were calculated. Notably, both compounds exhibited exceptionally high SI values of >1069.52 and >306.56 for PQM-243 and PQM-249, respectively (Table 1). These results underscore their strong antiviral potential, showing high efficacy against infection with reduced risk of cytotoxicity.

### 2.2. Molecular Modeling

**Figure 4:**
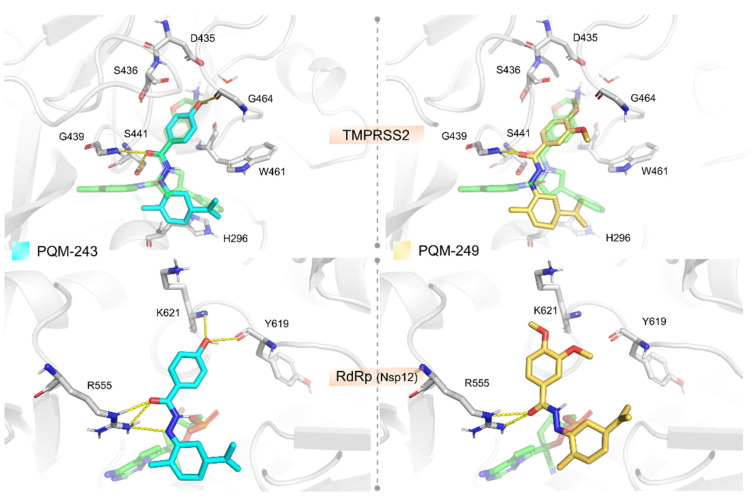
Docking results for compounds PQM-243 (**left**, cyan sticks) and PQM-249 (**right**, yellow sticks) against TMPRSS2 (**top**) and RdRp (**bottom**), showing key hydrogen bonds (yellow dashed lines). The reference compounds co-crystallized in the receptor structures (PDB codes 6T7P and 7BV2, respectively, for TMPRSS2 and RdRp) are represented as transparent green sticks.

We investigated the potential therapeutic targets of PQM-243 and PQM-249 through molecular docking to predict their binding modes and affinities (Table S1). When assessing docking results for some host-cell entry proteins (ACE2, Cathepsin B, and TMPRSS2) both compounds exhibited the most favorable binding affinities for TMPRSS2. In this case, the compounds were predicted to interact in a nanomolar range, with PQM-243 displaying slightly better predicted binding free energy (ΔG_bind_ = −8.862 kcal/mol) than PQM-249 (ΔG_bind_ = −8.502 kcal/mol). Docking analysis of PQM-243 and PQM-249 against the TMPRSS2 model revealed a well-defined binding model, similar to the co-crystallized ligand in the template structure (PDB code 6T7P) stabilized by key interactions within the receptor cavity (Figure 4). Their overall binding modes place the substituted phenyl moiety as closely filling the S1 pocket, the N-acyl-hydrazone (NAH) linker placed in the canonical oxyanion hole, and the terpene moiety near hydrophobic residues in the S1′ pocket. The NAH moiety of both compounds forms two H-bonds with the oxyanion hole (Gly184 and Ser186), suggesting potential inhibitory activity. The key difference between them lies in the substituent attached to the phenyl ring positioned in the S1 pocket, which may account for the slight variation in their predicted binding affinities. In PQM-243, the hydroxyl group can establish an H-bond with the backbone carbonyl of the conserved Gly219 residue. In contrast, PQM-249 features a more hydrophobic ether moiety in this region, interacting primarily through lipophilic contacts. In addition to TMPRSS2, both compounds were predicted to interact weaker, but still in the low micromolar range, with the RBD domain from Spike (−7.909 kcal/mol for PQM-243 against the RBD-ACE2 region and −7.473 for PQM-243 against the linoleic cavity) and the ACE2 active site (–7.866 kcal/mol for PQM-249 and −7.473 for PQM-243). Such findings suggest that the ACE2 and the RBD domain from Spike might act as secondary targets that these compounds could interact with to block the cell invasion process.

The docking experiments against the selected viral replication targets (i.e., Nsp3, Mpro, Nsp12, and Spike) indicated that both compounds exhibited the best-predicted affinity against the Nsp12 protein (PDB code 7BV2). Particularly, PQM-243 demonstrated a more favorable binding free energy in a nanomolar range, whereas PQM-249 was predicted as a low micromolar compound (ΔG_bind_ = −8.326 kcal/mol for PQM-243 and ΔG_bind_ = −7.948 kcal/mol for PQM-249, Figure 4).

### 2.3. Angiotensin Converting Enzyme-2 (ACE2) Inhibition Assays

Based on literature data indicating that CBD can prevent viral entry into host cells, we investigated a potential mechanism that could explain the inhibitory activity of PQM-243 and PQM-249 against SARS-CoV-2. Specifically, we assessed their ability to bind to the ACE2 receptor, potentially blocking viral attachment to the host cell. The tested compounds and their respective IC_50_ values are presented in Table 2. Notably, both compounds exhibited ACE2 inhibition in a low nanomolar range, surpassing the potency of DX600, a standard ACE2 inhibitor, which displayed activity in the micromolar range.

### 2.4. Inhibition of the Interaction Between the Receptor Binding Domain (RBD) and ACE2

**Figure 5:**
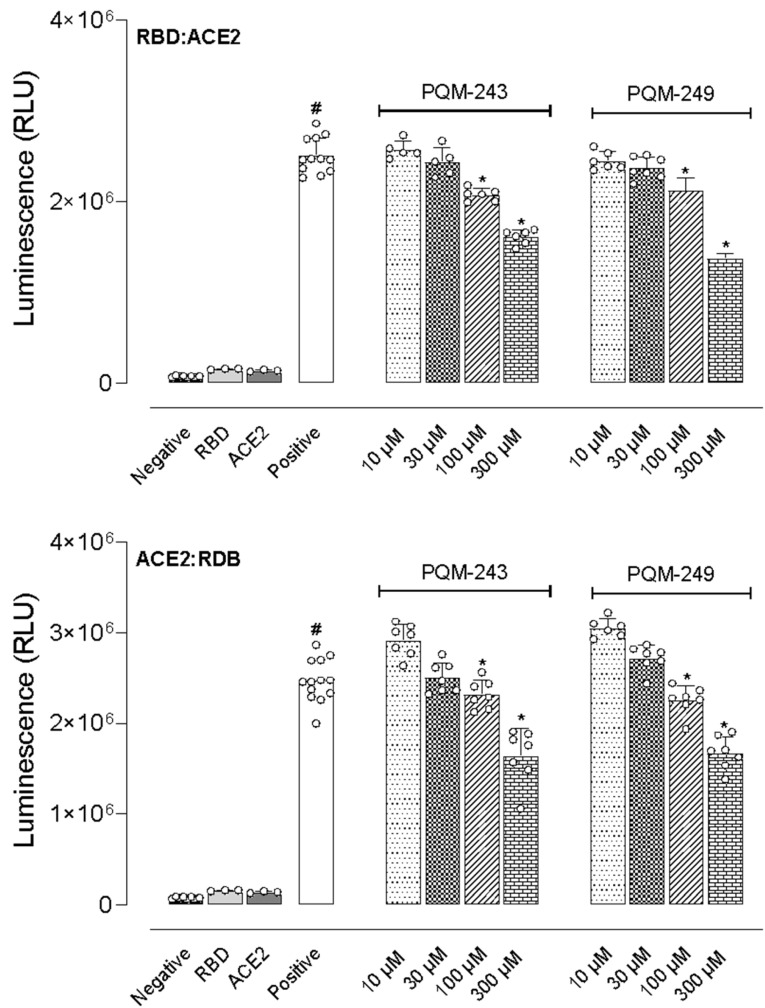
Detection of RBD:ACE2 interaction inhibitors with Lumit™ SARS-CoV-2 Spike RBD: hACE2 Immunoassay. PQM-243 or PQM-249 (at concentrations between 10 and 300 µM) were incubated with RBD prior to ACE2 addition (upper graph (RBD:ACE2) or PQMs were incubated with ACE2 prior to addition of RBD (lower graph (ACE2:RBD). Luminescence was recorded using a Varioskan equipment (Thermo Scientific Co., Waltham, MA, USA). RLU, relative unit of light. Values represent the mean of 7–13 replicates. Statistical analyses were done by ANOVA followed by Tukey’s post-test. # *p* < 0.05 indicates a statistical difference when comparing the positive group against the RBD or ACE2 groups. * *p* < 0.05 indicates a statistical difference when comparing different concentrations of each PQM against the positive group.

We also investigated whether the test compounds could interact with either the receptor binding domain (RBD) of the viral Spike glycoprotein homotrimers or the ACE2 receptors expressed by endothelial cells, ciliated bronchial epithelial cells, and type I and II pneumocytes. For this end, we employed two experimental protocols. In the first, different concentrations of PQM-243 and PQM-249 were incubated with RBD before the addition of ACE2. In the second, both compounds were first incubated with ACE2, followed by the addition of RBD. As shown in Figure 5, incubation of RBD with ACE2 (positive group) resulted in a 19.1-fold increase in luminescence compared to the negative control groups (RBD or ACE2 alone). Notably, both compounds significantly reduced the luminescence at concentrations above 100 μM, regardless of the protocol used. An antagonist effect was observed when compounds were pre-incubated with RBD before ACE2 addition, as well as when they were pre-incubated with ACE2 before the addition of RBD.

### 2.5. In Vivo Assays

**Figure 6:**
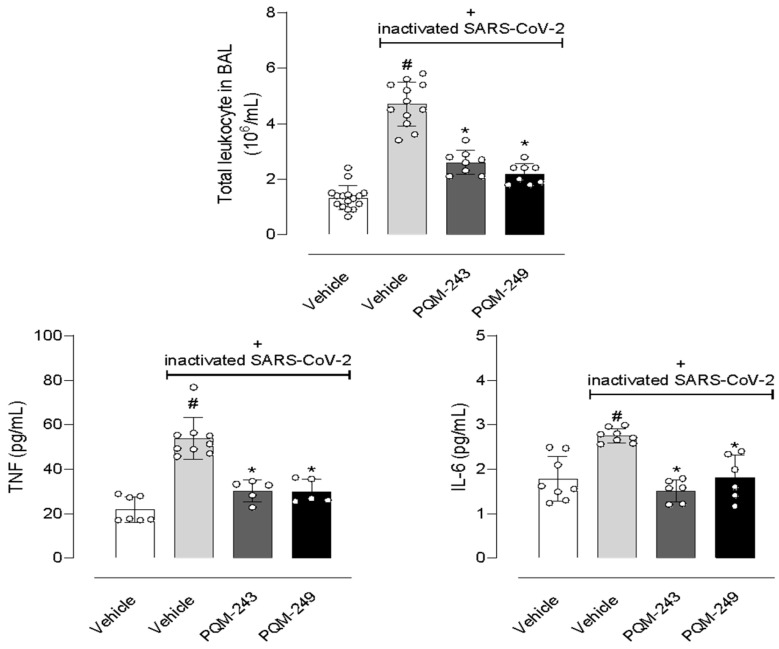
PQM-243 and PQM-249 reduce leukocyte infiltration and cytokine production in the bronchoalveolar lavage. Mice were treated with an oral dose of PQM-243 or PQM-249 (at 10 µmol/kg) at days 1, 3, and 5 post-nasal instillations of inactivated SARS-CoV-2 (10^5^ PFU). On the 7th day, mice were euthanized, and bronchoalveolar lavage was collected. Results are expressed as mean ± standard error (*n* = 5–16). Statistical analyses were done by ANOVA followed by Tukey’s post-test. # *p* < 0.05 indicates a statistical difference when comparing groups instilled with virus and pre-treated with vehicle to the group instilled with saline and pre-treated with vehicle. * *p* < 0.05 indicates a statistical difference when comparing groups instilled with virus and pre-treated with PQM-243 or PQM-249 to the group instilled with virus and pre-treated with vehicle. “+”: represent SARS-CoV-2 positive.

Considering the data obtained after incubation of PQM-243 and PQM-249 with SARS-CoV-2 or with virus-infected cells, we further evaluated the possible effects of both compounds in a model of lung pneumonia induced by the inactivated virus. Figure 6 shows that nasal instillation of inactivated SARS-CoV-2 led to a 3.6-fold increase in the leucocyte population in the lung bronchoalveolar lavage (BAL). It also led to 2.45- and 1.55-fold increased levels of the inflammatory cytokines TNF-α and IL-6, respectively, in the BAL. Oral pre-treatment of mice with PQM-243 or PQM-249, at a dose of 10 µmol/kg, significantly reduced leukocyte infiltration by 44.7% and 53.2%, respectively. Similarly, both compounds also reduced TNF-α levels by 44.4% and 44.4% and IL-6 by 46.4% and 35.7%, respectively.

**Figure 7:**
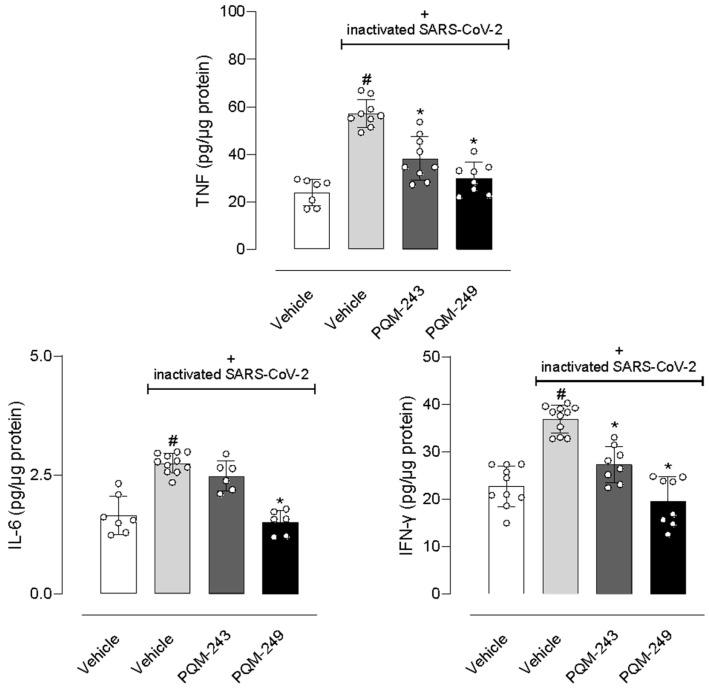
PQM-243 and PQM-249 reduced levels of cytokines produced in the lungs. Mice were treated with an oral dose of PQM-243 or PQM-249 (at 10 µmol/kg) at days 1, 3, and 5 post-nasal instillation of inactivated SARS-CoV-2 (10^5^ PFU). On the 7th day, mice were euthanized and lungs collected. The results are expressed as mean ± standard error (*n* = 6–11). Statistical analysis was performed by ANOVA, followed by Tukey’s post-test. # *p* < 0.05 indicates a statistical difference between the virus-instilled and infected vehicle-pre-treated groups with to the non-infected saline-treated and vehicle-pre-treated animal groups. * *p* < 0.05 indicates a statistical difference between the virus-instilled group and the infected group pre-treated with PQM-243 or PQM-249, with the virus-infected groups only treated with vehicle. PFU = plaque-forming units. “+”: represent SARS-CoV-2 positive.

Next, we focused on quantifying the key-inflammatory cytokines TNF-α, IL-6, and IFN-γ in lung tissue. First, nasal instillation of inactivated SARS-CoV-2 in mice resulted in a significant increase in levels of all three cytokines in the lungs. Our results showed a 2.3-, 1.64-, and 1.61-fold increase in TNF-α, IL-6, and IFN-γ levels, respectively. Notably, the treatment with PQM-243 and PQM-249 reduced TNF-α levels by 33.3% and 47.4%, respectively, and INF-γ by 27% and 45.9%, respectively, in lung tissue. In contrast, although both compounds significantly reduced cytokine levels, only PQM-249 affected IL-6 production, reducing its levels by 46.4% (Figure 7).

## 3. Discussion

Since the end of 2019, the severe acute respiratory syndrome coronavirus 2 (SARS-CoV-2), known as the COVID-19 pandemic, has caused impressive morbidity and mortality, with critical social and economic disruptions worldwide. Thus, the urgent need for alternative and effective treatments for this new pathological condition, in which none of the known available drugs showed clinical results, has led numerous academic and industrial research groups around the globe to start a race in the search for new treatment options.

In this work, we demonstrated that PQM-243 and PQM-249, two novel synthetic CBD-based terpene *N*-acyl-aryl-hydrazones, exhibited expressive dual antiviral and anti-inflammatory effects against SARS-CoV-2 infection. Additionally, computational studies suggested that these compounds were able to reduce direct virus interaction with both RBD and ACE2 proteins. Moreover, biological data were complemented by a pre-clinical animal model developed by our group, in which viral pneumonia was induced in mice by the instillation of inactivated SARS-CoV-2. In this model, both compounds exhibited a remarkable ability to reduce the virus-induced inflammatory condition.

In silico studies corroborated our biological data, suggesting that both compounds, PQM-243 and PQM-249, are able to interact at the entrance of the nucleotide channel to the TMPRSS2 and RdRp active sites, potentially blocking the influx of new nucleotides. Our analysis of the docking against RdRp showed that both compounds can form hydrogen bond interactions with Arg555, a key residue for stabilizing the incoming nucleotide in the right position for subsequent catalysis. Regarding their structure–activity relationship, the main difference between the two compounds appears to be the para-hydroxyl-substituent of PQM-243, which acts as an H-bond donor to the backbone carbonyl from Tyr619. In contrast, PQM-249 contains two ether substituents and was predicted to engage only in hydrophobic interactions in this region, potentially leading to less specific inhibition of the RdRp. These findings suggest that PQM-243 may be a promising candidate for further investigation, particularly in modulating TMPRSS2 and Nsp12 activities.

We further evaluated whether the compounds could directly interact and interfere with the virus life-cycle and viability, as well as their capacity to enter the human cell. Our results showed that both compounds exhibited a significant virucidal effect at a concentration of 100 μM. In the dose–response assay, both compounds exhibited nearly a 100-fold reduction in viral titer starting from the second-lowest concentration (1 μM). However, this effect was not dose-dependent, as the reduction was maintained across the higher concentrations tested. For PQM-243, even the 0.1 μM concentration produced a similar reduction in viral titer [5]. Indeed, more studies are required with different formulations for oral administration to determine the efficacy of these compounds in reducing viral load in vivo.

Several studies have addressed the relevance of the selectivity index (SI) as a crucial metric parameter to evaluate the relative antiviral efficacy and safety of compounds, since SI makes it possible to estimate the experimental window between cytotoxicity and antiviral activity of a given substance, and SI values ≥ 10 are suggested to be safer and more effective for treating a viral infection [52,53,54]. Notably, compounds PQM-243 and PQM-249 exhibited SI values much higher than 10, suggesting their relative safety regarding cytotoxicity.

Coronavirus entry into human cells is mediated by the interaction between the RBD receptor from the viral spike glycoprotein homotrimers and ACE2 receptors expressed by endothelial cells, ciliated bronchial epithelial cells, and type I and II pneumocytes. Since this interaction is essential for viral entry, it is, therefore, a promising therapeutic target for the development of novel neutralizing antibodies or small inhibitors that may interfere with its interaction with ACE2. In addition, neutralizing antibodies isolated from the plasma of convalescent patients block the binding of Spike protein RBD and ACE2, potentially conferring a protective effect. In this regard, we evaluated whether compounds could reduce ACE2 activity. Our data suggests that both compounds were at least 600-fold more potent than the ACE2 antagonist DX600, a commercially available ACE2 antagonist. This compound has already been reported to inhibit SARS-CoV-2 in vitro by William and colleagues [55]. However, DX600 is a highly selective peptide that has not been tested in vivo or in clinical regimens for its antiviral efficacy. In fact, this peptide forms multiple interactions with the catalytic site; however, its binding sites are distinct from the receptor-binding domain of the SARS-CoV-2 virus [55]. Also, it is not known if the binding site of DX600 would be, per se, sufficient to change the tridimensional conformation of ACE2, or induce a robust steric hindrance, in order to inhibit spike binding from within the catalytic site. Therefore, DX600 was only employed as a positive control for ACE2 inhibition on the enzymatic assay and not as a reference antiviral drug.

Most patients infected with SARS-CoV-2 have mild symptomatic manifestations, with no apparent symptoms or exhibiting a mild picture of a respiratory commitment or pneumonia. However, another group of approx. 14% of individuals develop a severe picture of the disease, including dyspnea, hypoxia, or lung injury, and 5% of patients exhibit a critical inflammatory condition, characterized by respiratory failure, systemic shock, or multiple organ failure [56]. After SARS-CoV-2 infection, a viral replication process is detected through pattern recognition receptors (PRRs), which include the family of Toll-like receptors (TLRs). These virus-specific structures culminate in the oligomerization of these receptors and the activation of transcription factors, mainly interferon regulatory factors (IRFs) and nuclear factor kB (NF-kB) [57]. Transcriptional activation of IRFs and NF-kB results in cellular antiviral defenses, which are mediated by the transcriptional induction of type I and III interferons (IFN-I and IFN-III), respectively, and subsequent positive regulation of IFN-stimulated genes (ISGs). However, SARS-CoV-2 exhibits several strategies to sabotage innate immunity, particularly the IFN-stimulated pathways [58]. Immunomodulatory therapies that allow for profuse production of IFN and modulation of other pro-inflammatory cytokines, such as TNF-α and IL-6, have been proposed as strategies for blocking SARS-CoV-2 and other respiratory viral infections [59]. Importantly, a second post-infection response involves the recruitment and coordination of specific leukocytes through the secretion of pro-inflammatory chemokines and cytokines [60]. Taken together, once early activated and properly located, these two immune defense systems can limit viral infection and progression [39].

As a part of an ongoing project aimed at the discovery of new easy-access synthetic compounds, with potential multifunctional properties against SARS-CoV-2 infection, two series of CBD-based terpene-*N*-acyl-hydrazones were investigated. As a result of preliminary in vitro screening, compounds PQM-243 and PQM-249 stood out for their virucidal effects, with no significant cytotoxicity on human cells. Next, to evaluate their potential anti-inflammatory effects in a COVID-19-like condition of bronchoalveolar injury, both compounds were submitted to an in vivo pre-clinical model of SARS-CoV-2-induced pneumonia. In this model, the animals were infected by inactivated-virus instillation, and after 7 days, they were euthanized, and their lungs were collected. Our data showed that both compounds, when used orally at a dose of 10 µmol/kg, significantly reduced leukocyte infiltration in the lungs, as well as cytokine production. Additionally, an expressive reduction in the cytokine levels in lung tissue was observed, and our experimental data corroborate with the literature regarding the increase in cytokine levels in virus-infected animals. It is worth noting that PQM-243 and PQM-249 were administered orally and, in addition to their virucidal effects, both exhibited significant effects on reducing pulmonary inflammatory patterns [61].

## 4. Materials and Methods

### 4.1. Synthesis of PQM-243 and PQM-249

The synthesis and characterization of the target compounds PQM-243 and PQM-249 were recently reported by our group [60,61]. In brief, the synthesis followed a three-step synthetic route, starting from commercially available 4-hydroxy- and 3,4-methoxybenzoic acids, which were interconverted into the corresponding acid chlorides and then to the respective benzohydrazides. Then, the key-benzoyl-hydrazides were coupled with *R*-carvone or *S*-carvone under acidic catalysis to furnish PQM-243 (40%) and PQM-249 (55%), respectively. The purified compounds were obtained as pale-yellow solids by removing the solvent and filtering the resulting solid with ice-cold methanol. All compounds were characterized by IR, NMR, and HRMS techniques [60].

### 4.2. Cell Lines and SARS-CoV-2 Cultures

Vero cells (African green monkey kidney, ATCC^®^ CCL-81™) were cultured and maintained in minimum essential medium (MEM) (Sigma-Aldrich, St. Louis, MO, USA), containing 5% fetal bovine serum (Cultilab, Brazil) and a cocktail of the antibiotics streptomycin (Sigma-Aldrich, USA [100 μg/mL]), potassium penicillin (Sigma-Aldrich, St. Louis, MO, USA [100 U/mL]), and amphotericin B (Bristol-Myers Squibb, Brazil, [2.5 μg/mL]). The cultures were incubated in a humidified oven at 5% CO_2_ and 37 °C.

The SARS-CoV-2 Wuhan culture was used in the antiviral activity tests in the biological safety level 3 laboratory (BSL-3) of the Institute of Biological Sciences, UFMG, Brazil. The virus was propagated in Vero monolayers in 175 cm^2^ culture flasks. Cells were infected at a multiplicity of infection (MOI) of 0.01 and incubated at 37 °C with 5% CO_2_ atmosphere for 72 h, or until a cytopathic effect was observed. The supernatant was collected and centrifuged (centrifuge MultifugeTM, X3R, Thermo Scientific, Waltham, MA, USA) for 15 min at 2500 rpm at 25 °C and stored at −80 °C until use. The viral titration was carried out using the Dulbecco plate assay as previously described [62,63,64].

### 4.3. Cytotoxicity Assay

Vero CCL-81 cells were plated in 96-well microplates (2 × 10^4^ cells/well). Next, the solutions of 100 μL/well of MEM with 1% SFB containing different compounds in a concentration range varying from 100 to 0.1 µM were added to the wells. In the same test plate, the cell controls, the cell death control (Triton X-100 10% *v*/*v*), and the vehicle control (DMSO 0.5% *v*/*v*) were included. The cells were incubated for 72 h at 37 °C and 5% CO_2_. After the incubation period, the supernatant was discarded, and the alamarBlue^TM^ (Thermo Fisher Scientific Co., Waltham, MA, USA) reagent (10%) was added and incubated at 37 °C with 5% CO_2_ for 4 h in the dark. All conditions were carried out in internal triplicate and reproduced in two independent experiments. The results were analyzed by reading the absorbance on a Multiskan Go spectrophotometer (Thermo Fisher Scientific Co., Waltham, MA, USA) at a wavelength of 577 nm and expressed in terms of the percentage of cell viability as previously described [62,63,64].

### 4.4. SARS-CoV-2 Virucidal and Dose–Response Assays

For the virucidal assay, 200 µL of the SARS-CoV-2 Wuhan was incubated with 200 µL of PQM-243 or PQM-249 (100 µM), MEM (control group), and DMSO (1%), in a final volume of 400 µL. These samples were incubated at 37 °C for 1 h, and the remaining infectious virus was titrated by plaque assay. For the dose–response assay, 1 × 10^5^ Vero CCL-81 cells/well were seeded and incubated for 24 h at 37 °C and 5% CO_2_. The following day, cells were counted in a hemocytometer, and an infection was performed with MOI 0.01 of SARS-CoV-2 Wuhan. After 1 h of adsorption, the viral inoculum was washed once with phosphate buffer saline (PBS) and the infected monolayers were covered with overlay media containing the test compounds as follows: (1) Virus positive control; (2) DMSO control; (3) PQM-243 (at 0.01, 0.025, 0.05, 0.1, 1, 10 and 100 µM); and (4) PQM-249 (at 0.01, 0.025, 0.05, 0.1, 1, 10 and 100 µM). Infected cells were submitted to treatment regimens for 48 h post-infection (HPI) when the supernatant was collected and titrated for infectivity using plaque assay. The curves for evaluating the antiviral dose–response activity of the compounds were plotted using the viral titer results, and the calculation of the absolute IC_50_ was based on the ideal non-linear regression for interpolating sigmoidal curves. The calculations were carried out using GraphPad Prism software (version 9.0) [62,63,64,65].

### 4.5. Molecular Modelling

The virtual screening experiments with compounds PQM-243 and PQM-249 against potential therapeutic targets related to COVID-19 were performed with the DockThor-VS platform [66,67,68]. For this study, we selected therapeutic targets related to host-cell entry (the substrate binding site of ACE2, CathB, and TMPRSS2) and viral replication (PLpro and macro domains of Nsp3, active site of Mpro, Nsp12, and Spike). For Spike, we performed docking experiments at two binding sites across four structures: the ACE2 interaction interface in the 6M0J structure (both free and complexed with ACE2), and the 7BZ5 structure (antibody-free), as well as the linoleic acid binding site in the 6ZP2 structure.

We used the curated receptor structures available in DockThor-VS Platform, as they had been previously prepared to optimize the hydrogen bond network and adjust the protonation and tautomeric states, considering the presence of the reference compounds (when available) [67]. As exceptions, the metal-binding site of ACE2 (PDB ID: 1R4L) [66] and the linoleic acid pocket of the Spike protein (PDB ID: 6ZP2) [69] were specifically prepared for this work. In such cases, the protein preparation was performed using the Protein Preparation Wizard in Maestro (Schrödinger Release 2025–1: Maestro, Schrödinger, LLC, New York, NY, USA, 2025). The assignment of protonation states and optimization of hydrogen bonds were carried out using PROPKA (version 2) [70], considering the experimental pH conditions and, when applicable, the presence of a co-crystallized ligand. The compounds PQM243–249 were also prepared in the Maestro suite using the classical version of LigPrep/Epik to predict the protonation and tautomeric states of the ionizable groups of the compounds [71,72]. A thorough visual inspection was conducted to verify the protonation and tautomeric states of the binding site residues and ligands. When necessary, adjustments were made based on information from the literature.

The DockThor program applies a grid-based methodology, where ligands remain flexible while the receptor is treated as rigid. The grid boxes used as the search space for each receptor were previously determined as described elsewhere. In the case of the metal-binding site of ACE2 and the linoleic acid pocket of Spike, the center of the search space was defined as the geometric center of their co-crystallized compound.

The DockThor pose prediction is performed through a multi-solution genetic algorithm alongside the MMFF94S molecular force field scoring function [73]. The virtual screening experiments were conducted using the standard mode of the genetic algorithm as follows: (i) 24 docking runs, (ii) 1,000,000 evaluations for each docking run, and (iii) a population of 750 individuals. To estimate binding affinity and rank protein–ligand complexes, we utilized DockTDeep, a convolutional neural network-based model recently developed by our research group [74].

### 4.6. Animals

K18 mice (25–30 g) were kindly donated by Central Mouse Bioterium from Health Science Center (UFRJ, Rio de Janeiro, Brazil) and maintained in the Animal Experimentation Unity in the Institute of Biomedical Science (UFRJ) in a room with a light–dark cycle of 12 h, 22 ± 2 °C, at 60–80% humidity, and with food and water provided ad libitum. The animals were acclimatized to the laboratory conditions for at least 1 h before each test on set and were used only once throughout the experiments. All protocols followed the principles and guidelines adopted by the National Council for the Control of Animal Experimentation (CONCEA), approved by the Ethical Committee for Animal Research (# 96/23). All experimental protocols were performed during the light phase. Animal numbers per group were kept at a minimum, and at the end of each experiment, mice were killed by a ketamine/xylazine overdose. In each assay, the animals were pre-treated by oral gavage with a dose of 10 µmol/kg of either PQM-243 or PQM-249 at 1, 3, and 5 days after virus instillation (as described below).

### 4.7. SARS-CoV-2-Induced Lung Inflammation

Both compounds were dissolved in dimethyl sulfoxide (DMSO) to prepare 100 µmol/mL stock solutions. Before their use, solutions were freshly prepared from each stock solution using saline (NaCl 0.9%). Doses of 10 µmol/kg (final volume of 0.1 mL per animal) were administered by oral gavage. The control group was treated only with vehicle. Mice were anesthetized with ketamine/xylazine and received an intranasal instillation of the inactivated virus (10^5^ PFU per mice). Animals were maintained in decubitus positions for 10 min and then were returned to the boxes. After 7 days, mice were euthanized with an overdose of ketamine (25 mg/kg)/xylazine (10 mg/kg) solution. A cannula was fixed in the trachea, and 1 mL of sterile PBS was injected and further collected. The bronchoalveolar lavage was centrifuged at 1500 rpm, 4 °C, 10 min. Supernatant was collected and stored at −80 °C for several measurements. Blood was collected in a tube with clot separator gel, and serum was collected and stored at −80 °C for several measurements. A group of mice was submitted to perfusion with saline (NaCl 0.9%) to remove all blood. The lungs were collected and frozen for cytokine and RT-PCR measurements.

### 4.8. Angiotensin Converting Enzyme-2 (ACE2) Enzyme Assay

To perform the assay, controls were composed by (1) assay buffer (Tris-HCl pH 7.4, 1 M; NaCl, 1 M; ZnCl_2_, 0.1 M), protein (1 mg, obtained from cell suspension) and water; (2) assay buffer, protein, ACE2 substrate (Mca-APK(Dnp), 1 µM), ACE2 inhibitor (DX600, 10 µM) and water. Sample tubes were composed of assay buffer, protein, ACE2 substrate (Mca-APK(Dnp), 1 µM), PQM-243 or PQM-249 (10, 30, 100, and 300 µM each compound), and water. After 30 min incubation at 37 °C fluorescence was measured at 380 nm excitation and 460 nm emission using a Varioskan plate reader (Thermo Scientific Co., Waltham, MA, USA). Enzyme activity was measured by the difference between the control groups and expressed as µM/min/mg of protein.

### 4.9. Interaction Between Spike Protein with Angiotensin Converting Enzyme-2 (ACE2)

It is a bioluminescence assay that measures the levels of interaction between the SARS-CoV-2 Spike protein binding domain (RBD) region with human angiotensin-converting enzyme 2 (ACE2). This assay combines immunodetection with NanoBiT (a structural complementation reporter ideal for studying protein–protein interactions (PPIs). When two proteins (one bound with LgBiT—large BiT and the other bound with SmBiT—small BiT) interact, the subunits are brought into proximity to form a functional enzyme that generates luminescence in the presence of its substrate. The protocol was used according to the kit manufacturer (Lummit SARS-CoV-2 RBD:ACE2 screening Immunoassay, Promega Corporation, Madison, WI, USA). Briefly, different concentrations of PQM-243 or PQM-249 were incubated with RBD and buffers, and after 30 min of incubation, ACE2 was added and incubated for further 60 min at room temperature. In another protocol, different concentrations of PQM-243 or PQM-249 (10, 30, 100, and 300 µM) were incubated with ACE2 and buffers, and after 30 min of incubation, RBD was added and incubated for a further 60 min at room temperature. Luminescence was measured in a luminometer (Varioskan, Thermo Fischer Scientific, Waltham, MA, USA).

### 4.10. Cytokine Measurements

Supernatants from the exudates collected from the BAL or lungs were used to measure the levels of the cytokine tumor necrosis factor (TNF), interleukin-6, and interferon-γ by enzyme-linked immunosorbent assay (ELISA) using the protocol supplied by the manufacturer (B&D, Franklin Lakes, NJ, USA).

### 4.11. Statistical Analyses

The results are presented as mean ± SD or standard error of the mean calculated using Prism Software 10.1.2 (GraphPad Software, La Jolla, CA, USA) with 5–7 animals per group. For in vitro assays, each protocol was repeated at least three times (on three different days), and each experimental group was carried out in duplicate. The IC_50_ (concentration of substances that resulted in 50% inhibition) values were calculated as previously described [52,53,54,55], and one-way or two-way analysis of variance (ANOVA), followed by Tukey’s or Kruskal–Wallis post hoc test, was performed. The post hoc tests were run only if F achieved the necessary level of statistical significance. When *p* was lower than 0.05, group differences were considered significant.

## 5. Conclusions

Taken together, our data suggests that PQM-243 and PQM-249—two newly synthesized, easily accessible, and structurally simplified CBD-based compounds—can act as potent direct anti-SARS-CoV-2 agents, capable of reducing both viral viability and host cell entry by inhibiting the interaction between the viral receptor-binding domain (RBD) and the ACE2 receptor. Notably, both compounds exhibited a significant multi-target-directed pharmacological profile, demonstrating not only virucidal activity but also the ability to reduce SARS-CoV-2-induced lung inflammation when administered orally. These findings support further investigation of PQM-243 and PQM-249 as promising prototypes for the development of innovative drug candidates targeting SARS-CoV-2 and other virus-related respiratory diseases.

## Figures and Tables

**Table 1:** Cytotoxicity and anti-SARS-CoV-2 activity of compounds PQM-243, PQM-249, and nirmatrelvir.

Compounds	CC_50_ ^a^ (µM)	IC_50_ ^b^ (µM)	Selectivity Index (SI) ^c^
PQM-243	>100	0.0648 ± 0.041	>1543.21
PQM-249	>100	0.2860 ± 0.057	>349.65
nirmatrelvir	>100	0.179	>558.66

^a^ Cytotoxic concentration for 50% of the cells. ^b^ Inhibitory concentration for 50% of viral particles. ^c^ Selectivity Index (SI): ratio between the CC_50_ and the IC_50_ of the compound evaluated.

**Table 2:** IC_50_ values against ACE2 for PQM-243 and PQM-249.

Compound	IC_50_ (nM)
PQM-243	12.1
PQM-249	13.3
DX600	8300

## Data Availability

The original contributions presented in this study are included in the article. Further inquiries can be directed to the corresponding author.

## Supplementary Materials

The following supporting information can be downloaded at: https://www.mdpi.com/article/10.3390/ph18101565/s1, Table S1: results docking.
