# Supplementary material for: Novel Multifunctional Cannabidiol-Based Analogues with In Silico, In Vitro, and In Vivo Anti-SARS-CoV-2 Effect
Source: Pharmaceuticals (Basel). 2025 Oct 16;18(10):1565. doi: 10.3390/ph18101565 (PMC12567437; doi:10.3390/ph18101565)
Supplement: Supplementary file 1 [file pharmaceuticals-18-01565-s001.zip › pharmaceuticals-3806148-supplementary.pdf]

Compound	ACE2 1R4L	CathB 6AY2 ionicPair	CathB 6AY2 neutral	TMPRSS2	Nsp3 6WX4 PLpro	Nsp3 6W02 Macro domain	Nsp5 6W63	Nsp12 7BV2	Spike 6m0j no ACE2	Spike 6m0j+ACE2	Spike 7bz5 no ACE2	Spike 6ZP2 Linoleic SIte
**PQM-243**	-7.473	-7.244	-6.157	**-8.862**	-7.839	-7.361	-7.128	-8.326	-6.577	-6.448	-7.909	-7.311
**PQM-249**	-7.866	-7.175	-7.245	**-8.502**	-6.995	-7.342	-7.810	-7.948	-6.335	-7.421	-6.548	-7.842
